# A decision aid is not the quick fix for improving shared decision-making in advanced Parkinson’s disease: results of a mixed methods feasibility study

**DOI:** 10.1007/s00415-025-12972-x

**Published:** 2025-03-13

**Authors:** Frouke A. P. Nijhuis, Bas Schippers, Bastiaan R. Bloem, Bart Post, Marjan J. Meinders

**Affiliations:** 1 Department of Neurology, Canisius Wilhelmina Hospital, Nijmegen, The Netherlands; 2 Department of Neurology, Center of Expertise for Parkinson and Movement Disorders, Research Institute for Medical Innovation, Radboud University Medical Center, Nijmegen, The Netherlands; 3 Department of Neurology, Center of Expertise for Parkinson and Movement Disorders, Donders Institute for Brain, Cognition and Behaviour, Radboud University Medical Center, Nijmegen, The Netherlands

**Keywords:** Shared decision-making, Parkinson’s disease, Feasibility study, Mixed methods design, Decision aid

## Abstract

**Background:**

Choosing a device-assisted treatment for persons with Parkinson’s disease (PwPD) is a complex decision. We developed a shared decision-making (SDM) intervention to facilitate this decision. In this study, we evaluate the feasibility of this intervention from the patients’ perspective.

**Methods:**

We performed a multi-center, mixed-methods feasibility study with an uncontrolled pre-post-intervention design. Five neurologists and seven Parkinson nurse specialists from five Dutch hospitals participated. We aimed to enroll 20 PwPD in the usual-care group receiving decision support as usual, and 20 PwPD receiving the SDM intervention. The intervention consisted of a patient decision aid and a training for professionals. We evaluated feasibility by measuring acceptability, level of implementation, efficacy and the study procedures. For data collection, we used questionnaires, interviews, cognitive testing, consultation recordings, fieldnotes, and usage of the patient decision aid.

**Results:**

We included 19 PwPD in the usual-care group and 13 in the intervention group. Acceptability was good and implementation levels at the patient level were adequate: 92% of the participants used the patient decision aid, of which 77% the website and 69% the value clarification tool. The intervention improved PwPD’s knowledge on treatment options, however, it did not improve SDM. The SDM intervention was not used as intended and the initial treatment preference of either the PwPD or the professional directed the information exchange.

**Conclusions:**

Inclusion of PwPD for the study was limited. Acceptability of the SDM intervention was good, however, the patient decision aid should be used in collaboration between physicians and patients to enhance SDM.

**Trial registration:**

NTR6649, registered 28–08–2017 (available through ICTRP search portal).

**Supplementary Information:**

The online version contains supplementary material available at 10.1007/s00415-025-12972-x.

## Introduction

In the advanced stages of Parkinson’s disease (PD) disability accumulates due to a range of motor and non-motor symptoms. This advanced disease stage is also typically characterized by response fluctuations that are increasingly being perceived as disabling. Deep brain stimulation (DBS), levodopa-carbidopa intestinal gel (LCIG) or continuous subcutaneous apomorphine infusion (CSAI) are available treatment options to reduce these response fluctuations and thereby improve quality of life [1]. Ideally, in a shared decision-making (SDM) process, the decision for one of the treatment options is tailored to the person’s clinical characteristics and personal preferences. However, in current practice, SDM application is suboptimal [2].

SDM is a decision-making process that consists of four steps: the professional informs the patient that a decision needs to be made and that the patient’s opinion is important, the professional explains the options with the pros and cons of each option, the professional and patient deliberate together on the options and the preference of the patient and discuss which role the patient prefers in the decision-process, ending with making or deferring the decision, and possible follow-up[3]. Patient decision aids are widely used to improve SDM [4]. Patient decision aids are useful tools for both patients and healthcare providers, providing them with (1) necessary knowledge, (2) value clarification, (3) and guidance in deliberation (careful consideration and exploration of options and personal preferences), and communication in the decision-making process. Patient decision aids have the potential to improve the SDM process and to lower decisional conflict [4]. Despite the positive effects of patient decision aids found in clinical trials, only a fraction of these decision aids is implemented in routine clinical practice [5]. Barriers to the implementation of such patient decision aids include clinicians’ indifference to use it, lack of an implementation plan, and lack of funding [5, 6]. The patient perspective on facilitators and barriers for SDM revealed both modifiable and unmodifiable factors in (1) how the healthcare system is organized and (2) what happens during the decision-making interaction [7]. However, there is scarce evidence on the patient perspective of barriers and facilitators specifically with respect to the implementation of patient decision aids or SDM interventions [5]. The gap between the positive effects of patient decision aids in studies and the limited implementation in routine clinical practice, and the notion that the barriers and facilitators for implementation lack a patient perspective, highlights the need to not only conduct trials to test the efficacy of a patient decision aid, but also to evaluate its feasibility of implementation and to include the patients’ perspective.

To prepare for a large clinical trial, we assessed in daily clinical practice the feasibility of implementing a SDM intervention to support the choice for a device assisted treatment in PD, from the perspective of persons with PD. The SDM intervention consisted of a patient decision aid and a one-hour training for professionals on SDM and how to use the SDM intervention. The evaluation (1) analyzed the acceptability of the intervention; (2) assessed the level of implementation; (3) tested the efficacy on a small-scale; and (4) evaluated study procedures.

## Methods

### Study design

This feasibility study had a multi-center, uncontrolled pre-post intervention, mixed methods design. The pre-post intervention design created the possibility for the participating professionals to reflect on the decision-making process with and without the SDM intervention. The full protocol is described elsewhere [8]. Here, we will report on the outcomes from the perspective of persons with PD (PwPD).

### Participants

#### Professionals

Neurologists were eligible if they (1) considered at least five PwPD per year for a device assisted treatment as part of the regular clinical practice; and (2) collaborated with a PD nurse specialist in the same hospital. We aimed for neurologists from five hospitals. Moreover, we aimed to include professionals with different levels of expertise with respect to the device assisted treatment options. Based on these criteria, we included five neurologists and seven PD nurse specialists from the five outpatient neurology clinics in the Netherlands. These clinics included academic medical centers (*n* = 2), teaching hospitals (*n* = 2), and a community hospital (*n* = 1).

#### Patients

We aimed to include 40 participants in total: 20 in the pre-intervention group (usual care group) and 20 in the post-intervention group (intervention group), following recommended sample sizes for pilot or feasibility studies [9–11]. PwPD were included in a convenience sample. A convenience sample is a type of non/probability sampling advised in limited efficacy testing in feasibility studies [12]. Participants were eligible if they: (1) were diagnosed with PD and considered to be a suitable candidate for device assisted treatment, as judged by their own neurologist; and (2) were eligible for all three treatment options at the beginning of the decision-making process.

### The SDM intervention

**Fig. 1:**
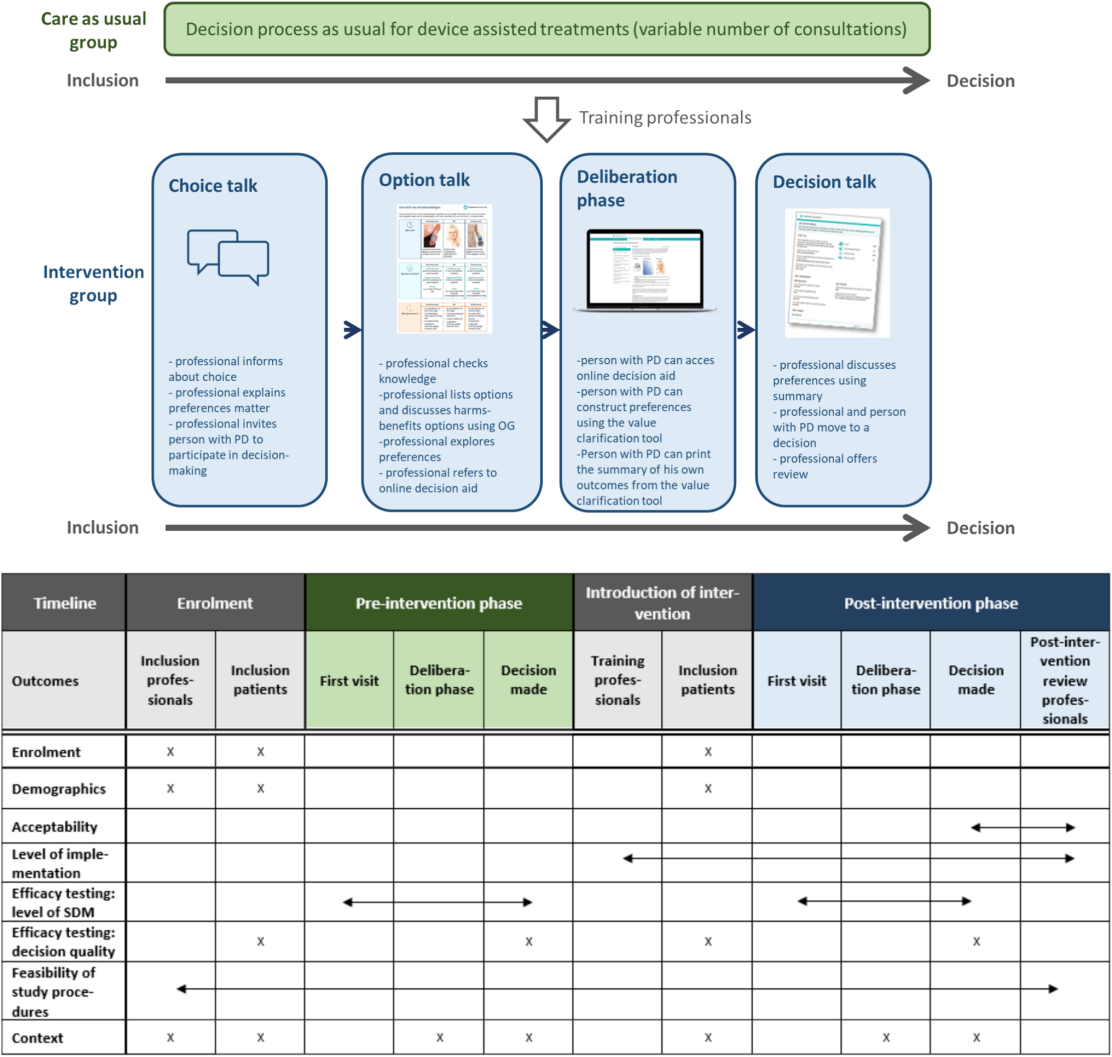
Decision process and data collection scheme (green represents the care as usual group, blue represents the intervention group)

The intervention has been developed following the process map for web-based decision support interventions [13]. The patient decision aid consisted of an Option grid™ (Supplement 2), and an online supplementary information website with a value clarification tool (complete patient decision aid via DOI in supplement 1). An Option grid™ is a one-page, evidence-based summary of available options presented in a tabulated format, listing the frequently asked questions that patients consider when making treatment decisions. An Option grid™ is meant to stimulate the discussion on treatment options in the clinical encounter [14]. The online website also contained the same Option grid™ but with supplementary information on all treatment options and the value clarification tool. A value clarification tool helps a patient to think about which attributes related to the available options matter to him most, to identify which option fits best his personal life [15]. The intended decision process in the post-intervention group would be (Fig. 1); the neurologist explains in the first encounter to the person with PD there is a choice of new treatment options to consider, and the patient’s preferences are important (choice talk). The key element of the SDM intervention was the Option grid™, to be used in the clinical encounter between the neurologist or PD nurse specialist to start the conversation about the treatment options with the benefits and harms of each option and to explore the patient’s preferences (option talk). After this first encounter, the person with PD was referred to the online version of the patient decision aid for more information about each treatment option and the value clarification tool (deliberation phase). In the following consultation, the summary of the patient’s value clarification exercise should be used to discuss the patient’s preferences and remaining questions on the treatments either by the PD nurse specialist or the neurologist. Together, they move towards a decision (decision talk).

The main researcher (FN) provided an on-site one-hour interactive training to the participating neurologists and PD nurse specialists on SDM and how to use the patient decision aid. Details of the intervention are described in the published study protocol [8].

### Study procedures

PwPD, found to be eligible for a device assisted treatment by the neurologist, were invited to participate in the study. The study team provided interested patients with information about the study and obtained written informed consent.

In the usual care group, patients received information and decision support as usual (Fig. 1). For the evaluation, we defined the end of the decision-making process as the moment that a choice for a treatment had been made and the screening for treatment eligibility was initiated. Once 20 PwPD in the usual care group had completed their decision process, the professionals participated in the one-hour training and they all received a personal login code for the online decision aid. Subsequently, PwPD for the intervention group were included and went through the decision process with the SDM intervention (Fig. 1). Once persons in the intervention group were included and had been introduced to the option grid by the neurologist or PD nurse specialist, they received a unique login code for the online decision aid as well.

### Outcome measures and data collection

This feasibility study focused on four aspects: acceptability, level of implementation, small-scale efficacy testing, and evaluation of study procedures, each with their own relevant outcome measures and methods of data collection (Table 1). In order to increase our understanding of the data outcomes, contextual factors were measured, based on our previous findings [2] (Table 1). A detailed description of the outcome measures and data collection methods is provided in the published protocol [8]. The data collection scheme is provided (Fig. 1).

**Table 1:** Research objectives, outcomes and measurement instruments

Objectives	Outcomes	Methods and instruments
Demographics	Age, gender, employment, living situation, education, disease duration, self-scored H&Y	Baseline questionnaire demographics for patients (H&Y scored by neurologist or PD nurse specialist)
Acceptability	Satisfaction with intervention and how intervention is received with all participating PwPD in the post-intervention group only	Structured questionnaires on readability, comprehensiveness, layout and information [16] interviews on perceived satisfaction, and perceived strengths and weaknesses of the intervention
Level of implementation	To what extent is the intervention implemented as planned	Field notes
	What proportion of the included participants use the intervention To what extent were all components of the intervention used	Audiotapes consultations, logging data of navigation behavior website, hard copies of value clarification tool summary: evaluation whether all elements of intervention are actually used
	How did participants react to the specific aspects of the intervention and to what extent did PwPD engage in the intervention	Analysis of audiotapes consultations, logging data of navigation behavior website to analyze the level of engagement of PwPD to all elements of the intervention Interviews on the interaction with the different intervention elements
Efficacy: level of SDM	Perceived level of SDM	Patient questionnaires: SDMQ-9 [17], CollaboRATE [18], CPS [19]
	Observed level of SDM	Researcher’s analysis of the consultations: OPTION-5 [20]
Efficacy: decision quality	Level of informed choice	Measuring knowledge in PwPD at start and end of decision
	Decisional conflict in decision making	Structured questionnaires for PwPD: DCS [21]
Evaluation of study procedures	Recruitment	Field notes on inclusion and drop-out rates
	Potential outcome indicators	Analysis of outcome measures from the efficacy testing with evaluation of conflicting data on outcome measures
	Approaches for data collection	Interviews with PwPD on acceptability of the study procedures Field notes on data collection
Context	Patient, professional or organization related factors in the implementation and outcomes	Questionnaires with PwPD with items on preferred role in decision making (CPS), treatment preference, pre-knowledge, health literacy skills (FCHHL), mood (HADS) Cognitive testing with PwPD using: MoCA Interviews with PwPD on implementation barriers and facilitators Audiotapes of consultations Field notes

*DCS* Decisional Conflict Scale, *CPS* control Preference Scale, *FCCHL* Functional Communicative and Critical Health Literacy, *HADS* Hospital Anxiety and Depression Scale, *MoCA* Montreal Cognitive Assessment

### Data analysis

Socio-demographic and disease-related data are presented descriptively as medians with interquartile range (IQR) or as frequencies with percentages. We performed a Mann–Whitney *U* test (continuous variables) or a Fisher exact test/Fisher-Freeman-Halton exact test (categorical variables) to statistically test differences in baseline characteristics, SDM-Q-9, CollaboRATE, CPS, knowledge test and DCS scores between the pre- and post-intervention groups. All quantitative statistical analyzes were performed in SPSS (Statistical Package for Social Sciences, Chicago, IL, USA).

For the qualitative analysis of the interviews, two independent researchers (FN and BS) analyzed the interviews based on the principles of thematic analysis [22]. All audio-recordings were transcribed verbatim. FN and BS read and re-read the transcripts and independently coded two interviews. Codes were compared and discussed during a consensus meeting until both researchers agreed on one set of codes. This code list was subsequently utilized to code two to four transcripts independently after which the researchers met and updated the code list by adding, merging and modifying codes. This process was repeated until all interviews were coded. Once coding was completed, we developed candidate themes and subthemes by merging codes that formed patterns in the data. Candidate themes and included codes were then iteratively reviewed and revised to ensure that themes and codes were representative for the data. Differences in interpretation during this process were discussed until we reached consensus on a final set of themes and sub-themes. The collation of codes into themes and sub-themes was conducted separately for the interviews from the pre- and post-intervention group and later cross validated again. This approach allowed us to analyze important aspects within groups as well as associations or discrepancies between (sub-)themes in both groups. The qualitative analysis was performed in ATLAS.ti (ATLAS.ti Scientific Software Development GmbH, Berlin, Germany). Quotes represented in the results were translated from Dutch. The data gained from the qualitative analysis, gave a deeper understanding to the quantitative outcomes. The qualitative data are represented in the tables under the heading qualitative data. The qualitative themes will be explicitly stated in the results and quotes given in the text to give examples are also from these data analysis, as well as the barriers and facilitators.

For the quantitative analysis of the consultations, the main researcher (FN), applied the five item “observing patient involvement” (OPTION-5) instrument. With this instrument, she scored the extent to which the clinician involved the patient in the decision-making process during consultations, according to the scoring manual [23]. This instrument is derived from the OPTION-12 instrument and assesses SDM behavior. The five items (scoring 0–4) comprise: 1, option talk (alternate options); 2, team talk (support deliberation); 3, option talk (information about options); 4, decision talk (eliciting preferences); and 5, decision talk (integrating preferences). If multiple consultations were necessary to reach a treatment decision, subsequent consultations were combined, and scored as one consultation. No additional consultations were scheduled for the study and followed normal consultation frequencies, but in the intervention group the consultation itself was more structured in steps (Fig. 1). Furthermore, notes were taken and qualitatively analyzed along the scoring of the OPTION-5 instrument. In the post-intervention group, the researcher also noted if and how the SDM intervention was used in the consultations.

#### Mixed methods analysis

The aim of the mixed method analysis was to assess the feasibility of the SDM intervention. To know if the SDM intervention is feasible to implement in clinical practice, part of feasibility is limited efficacy testing and knowing what has caused the effect. Therefore, it is important to explore how the SDM intervention actually influenced the decision process, creating a multidimensional understanding of the possible mechanisms of impact of the intervention on the outcomes [24, 25]. We applied both data triangulation and methodological triangulation. Data triangulation comprised the combination of data from the patient’s perspective and the researcher’s observations. Methodological triangulation was achieved by combining different methods of data collection, e.g., questionnaires, observations, interviews, field notes, and tracking logging behavior, to measure the same construct. For the analysis, we sorted the data, compared those data that measured the same outcomes based on different methodology, analyzed the level of agreement or dissonance and evaluated complementarity and divergence. Complementarity analysis means exploring whether different findings together explain a phenomenon or outcome and divergence reflects a disagreement between results from different sources. Each of these contributes to the validity of the research findings [34]. After analyzing all data, results were presented to the research group (all authors). Based on a group discussion, the researchers worked towards agreement on the findings and their interpretation.

## Results

We included five neurologists and seven PD nurse specialists who collaborated with these five neurologists, and who worked in five different hospitals in the Netherlands. They included a total of 34 PwPD: 20 in the usual care group during a period of 11 months, and 14 in the intervention group during an inclusion period of 24 months. One participant in the usual care group withdrew from the study during data collection due to personal reasons. One participant in the intervention group withdrew consent immediately after inclusion due to family circumstances. Both persons were excluded from the analysis. One center included PwPD only in the usual care group, despite several attempts to stimulate inclusion in the intervention group. The study was terminated prematurely as inclusion was not progressing despite several approaches to boost recruitment.

The evaluation interviews were successfully conducted with all PwPD. However, one recording in the usual care group was lost due to a corrupted audio file. Another interview (intervention group) was incorrectly recorded which was noticed directly after concluding the interview. The interviewer (FN) immediately summarized the person’s responses upon notification and sent the transcript to the participant for verification. The approved transcript was included in the analysis, resulting in a total of 31 interviews for the qualitative analysis. The average duration of the recorded interviews was 36 min for the pre-intervention group (*n* = 18) and 41 min for the post-intervention group (*n* = 13).

Audiotapes of the consultations were available for 16 (out of 19) PwPD of the usual care group and 8 (out of 13) of the intervention group. The other consultations were not recorded correctly and could therefore not be analyzed.

Socio-demographic and disease-related characteristics are summarized in Table 2. All baseline characteristics were comparable between both groups and did not differ statistically significant. Results of the MoCA cognitive tests showed that almost half of the PwPD suffered from (mild) cognitive impairment (MoCa score < 26) in both the usual care and intervention group.

**Table 2:** Socio-demographic and disease-related characteristics for the usual care and intervention groups

Demographics		Usual care	Intervention	*p*-value
Sample size (*n*)		19	13	
Gender [men, *n* (%)]		12 (63)	12 (92)	0.101
Age [median (range)]		64 (54–69)	65 (50–67)	0.623
Living situation [*n* (%)]	Alone	2 (11)	2 (15)	1.000
	Partner	15 (79)	11 (85)	
	Nursing home	1 (5)	0	
	Other	1 (5)	0	
Employment [*n* (%)]	Full time	2 (11)	0	0.753
	Part time	1 (5)	1 (8)	
	Unemployed	16 (84)	12 (92)	
Educational level [*n* (%)]	Primary school	3 (16)	0	0.508
	Secondary school	5 (27)	2 (15)	
	Lower vocational education	8 (42)	7 (54)	
	Higher vocational education	2 (10)	2 (15)	
	University	1 (5)	2 (15)	
Disease duration [*n* (%)]	< 5 years	3 (16)	1 (8)	0.757
	5–10 years	12 (63)	10 (77)	
	> 10 years	4 (21)	2 (15)	
H&Y [*n* (%)]	1	0	0	0.295
	2	7 (37)	7 (54)	
	3	11 (58)	4 (31)	
	4	1 (5)	2 (15)	
	5	0	0	
MoCA score [median (range)]		25.5 (19–30)*	27 (20–30)	0.489
MoCA score < 26 [*n* (%)]		9 (50)*	6 (46)	1.000

*MoCA* Montreal Cognitive Assessment, *H&Y* Hoehn and Yahr

*One participant did not want to undergo the cognitive tests

### Acceptability

Twelve out of the 13 (92%) PwPD from the intervention group had seen the Option grid™ and/or website. Overall, 10 of these 12 participants (83%) rated the amount of information as sufficient, while two persons found the information too limited. The overall explanation of the different treatment options was perceived as good to excellent for the majority (10/12, 83%). Of all the PwPD using Option grid™ and/or website, 10/12 (83%) said they would use them again in their decision process (Table 3 and Supplement 3 Table S1). The majority of PwPD reported in the interviews that the representation of risks and effects was hard to understand or confrontational to see. However, most understood the need to know those treatment aspects to fully grasp the implications of the treatment and create realistic expectations.

**Table 3:** Joint display of quantitative and qualitative data on acceptability of the SDM intervention

Quantitative data acceptability	Qualitative data acceptability
10/12 (83%) rated the amount of information as sufficient, 2/12 found the information too limited	PwPD who stated that they were satisfied with the Option grid and website, commented that the information was understandable and represented in a clear and structured way
Majority of patients found the information on effects, risks, daily care and follow-up procedure good to excellent. Patients scored the information on experience of other patients less but still 11/12 patients scored this fair to excellent	*“It [Option grid and website] is all pretty clear. The options, the success rate, the side effects and the risks are all systematically represented. That was very useful.” (67 yrs, M)* *“I think that everything that should be mentioned is incorporated [on the Option grid and website]. It is a clear story. There is nothing, in my opinion, no additional information that is not necessary.” (65 yrs, M)*
	One of the two patients who found the information too limited said “more information is always better”. And the other patient would have liked more personal advice based on symptoms
11/12 (92%) felt that the information was represented equally for all treatments, 1/12 (8%) felt it was geared towards DBS	The majority of patients reported that the representation of risks and effects was hard or confrontational to see. However, most understood the need to know those treatment aspects for the purpose of comprehensiveness of the intervention and providing realistic expectations
12/12 (100%) rated readability as “good”	Some patients had trouble understanding the representation of treatment effects and risks. This led one patient to believe that the represented numbers were based on studies with 100 patients while another interpreted no improvement in quality of life inherently as a decrease
	*“[…] the effects and risks, that scared me, thinking ‘76% chance at skin infections and all that stuff.’ Well, you should be realistic in knowing that you will not always have a good result and that is what it [Option grid] states.” (65 yrs, M)*
11/12 (92%) found the images to be clear 9/12 (75%) found the images to be of added value	
10/12 (83%) of the patients who used the PDA would use them again in their decision	Reason not to use PDA again: *“the Option grid/Website should lead to a personal advice which treatment would be more suitable for me based on my symptoms” (65 yrs, F)*
11/12 (92%) found that the value clarification tool made the decision easier, 1/12 felt it made the decision more difficult	*“It helped to make a deliberate decision” (50 yrs, M)*
	The single person who found it more difficult did not have a clear reason why it made it more difficult to choose

*PDA* patient decision aid

## Implementation

Implementation was measured on several levels (Table 1). The inclusion rates were limited. Different reasons were given (Table 4). Once included, all but one person was offered the Option grid™ and login code for the website by the neurologist/PD nurse specialist. Therefore, reach for the targeted users was good for included participants. The extent of implementation of the different components of the decision aid was satisfactory. The navigation behavior of the website showed that ten participants from the intervention group utilized the online information (10/13, 77%). Three PwPD viewed the information pages on all treatments almost equally long, one participant only visited pages of one treatment, two persons visited the pages of two treatments, and four persons, although visiting multiple treatment pages, clearly focused only on one or two of the treatments (Supplement 3 Table S2). All but one used the value clarification tool (9/10, 90%). The summary of the value clarification tool was discussed by 4 of the 9 participants who completed the tool with their neurologist and/or PD nurse specialist (Table 4). The paper-based Option grid™, the key element of the intervention, was used in a consultation by 12 out of 13 patients (Table 4 and Supplement 3 Table S2). The Option grid™ was discussed in the consultation of four PwPD. The Option grid™ was handed out to the other eight PwPD, used in the decision process to variable degrees by PwPD and both in and outside the consultations (Supplement 3 Table S2).

**Table 4:** Joint display of quantitative and qualitative data on implementation of the SDM intervention

Quantitative data	Interviews	Field notes	Data logging behavior	Audio consultation
7 hospitals invited, 5 hospitals included All hospitals included PwPD in the pre-intervention group 4 hospitals included PwPD in the post-intervention group Of each hospital at least one neurologist and at least one PD nurse specialist attended the training		One hospital did not include any PwPD in the intervention group. The neurologist explained that it was due to being understaffed, but also used a copied version of the decision aid outside the study In two hospitals, PD nurse specialists were understaffed		
**Inclusion of patients** Inclusion period pre-intervention: March 2015-April 2016 Inclusion period post-intervention: October 2017-May 2020 (early termination due to difficult recruitment)		Recruitment of eligible persons was the main barrier, once identified as eligible, most of them participated		
Eligible persons in pre-intervention group: 21 eligible, 20 included, 1 withdrawn Eligible persons in post-intervention group: 17 eligible, 14 included, 1 withdrawn				
**Use of PDA** OG: 12/13 Online PDA: 10/13 Value clarification tool online: 9/10	**Use of PDA** 1. OG is used as summary instead of discussion 2. Participant uses OG and online PDA to gain more information on preferred treatment 3. OG and online PDA prepare participant for consultation 4. OG and online PDA create more realistic expectations 5. Participant is able to corroborate decision through OG and online PDA	More nationwide attention for shared decision making More focus nationally for all three treatments and the healthcare logistics	3/10 viewed pages on all treatments equally	5/8 the OG was mentioned during the consultation
				2/8 the OG was discussed during consultation
4/9 discussed the summary of the value clarification tool with their neurologist and/or PD nurse specialist		Two hospitals copied the PDA in a paper version and used that one instead of the online one		
	**Facilitators to use PDA** 1. Easy access to information on the OG 2. Tear pad OG provides PwPD with the opportunity to read printed version 3. OG and online PDA creates opportunity to discuss information on options with partner and others			
	**Barriers to use PDA** 1. Participant is unable to access online components in an easy way			

*PDA* patient decision aid, *OG* Option Grid™

### Efficacy of the SDM intervention—level of SDM

In the usual care group, most PwPD preferred making the decision in a shared manner, while most PwPD in the intervention group preferred making the final decision themselves, while considering their neurologist’s opinion (Supplement 3 table S4). In reality, in both groups, most participants experienced that they made the decision themselves while considering their neurologist’s opinion. In the usual care group, in 10/19 (53%), there was discordance between the preferred and experienced roles, of which 6/10 (60%) would have preferred a less active role. For the intervention group, 9/13 (69%) experienced a different role than preferred, of which 6/9 (67%) would have preferred a less active role. Neurologists predicted the treatment preference of the PwPD correctly more often in the intervention group (100% vs 63%, *p* = 0.019) (Table 5).

**Table 5:**
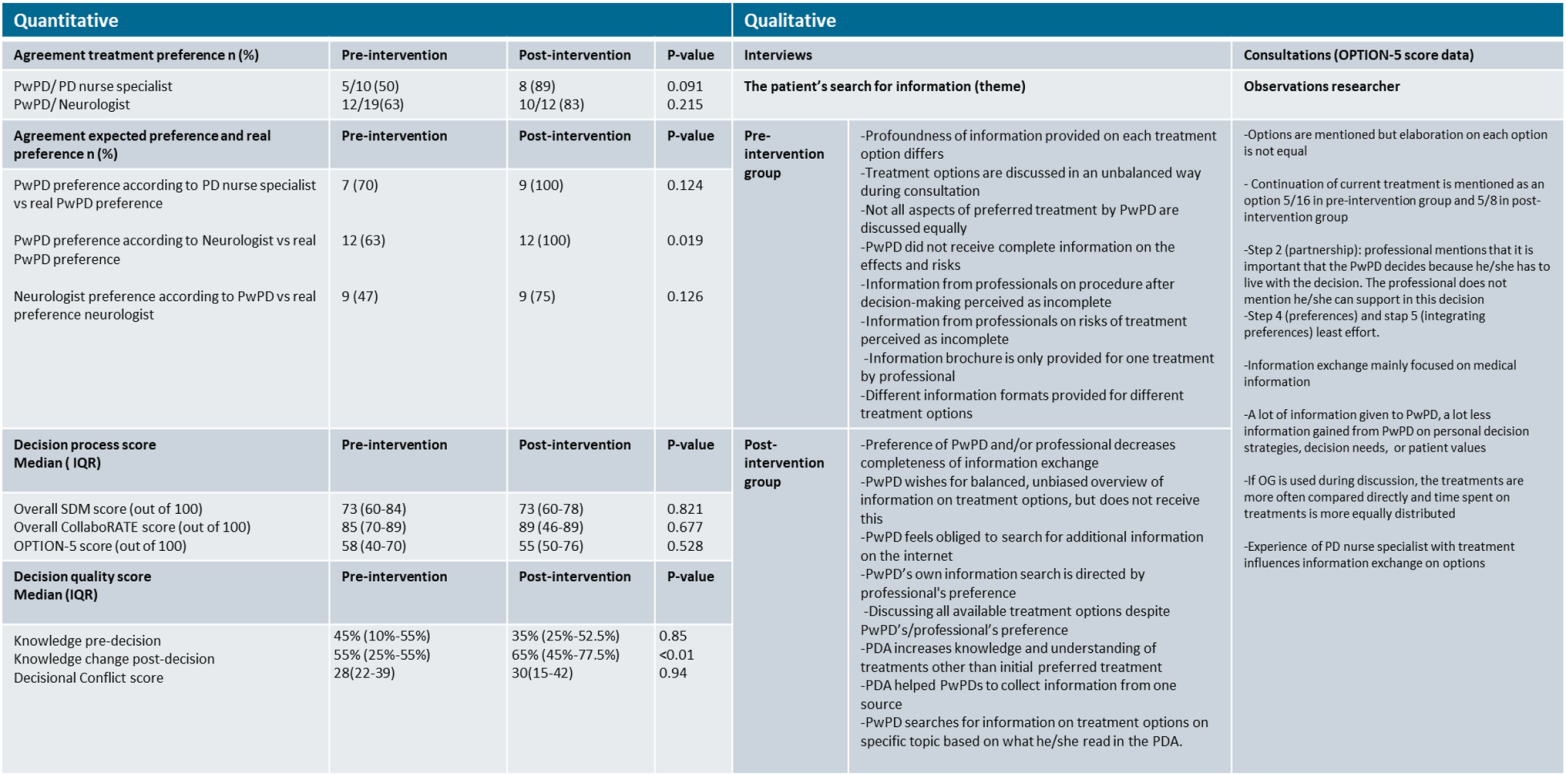
Joint display of quantitative and qualitative data on efficacy of the SDM intervention

*PDA* patient decision aid, *OG* Option grid™

The level of SDM did not differ in the intervention group compared to the usual care group, confirmed in the methodological triangulation. PwPD in the intervention group did not report higher levels of perceived SDM compared to the usual care group based on the SDM-Q-9 questionnaire (mean score of 73 out of 100, in both groups, *p* = 0.821) (Table 5, Supplement 3 Table S5). The median overall CollaboRATE score in both groups was high (Table 5), with no differences between the two groups (89 vs 85, *p* = 0.677). The observed SDM levels, measured in the OPTION 5 scores did not differ amongst the two groups (58 vs 55, *p* = 0.528).

### Efficacy of the SDM intervention—decision quality

A higher percentage of PwPD in the intervention group knew all device-assisted treatment options in comparison to the usual care group, but there was no statistically significant difference (77% vs 53%, *p* = 0.267). The intervention group performed significantly better than the usual care group on the knowledge test after making a decision (65% vs 55%, *p* < 0.01). The quality of the decision making did not differ between the groups (Supplement 3 Table S6).

### Evaluation of study procedures

The largest limitation was the inclusion rate. Reasons for limited inclusion were the understaffing of PD nurse specialists, the alternative use of a paper copy of the decision aid, and patients considered not eligible for all treatments. Recordings of consultations were limited, during the study we tried to improve this by supplying persons with PD with a personal recording device apart from the recording devices of the professionals.

### Mechanisms of impact

The mechanisms of impact are mechanisms through which the intervention activities produce intended or unintended effects. This can be evaluated by measuring the participants, interactions with the intervention and measuring intermediate process that explain changes in outcomes. Here, we describe two observed mechanisms of impact.

#### Gaining knowledge on treatment options

Within the qualitative theme *the patient’s search for information*, we observed that PwPD from the usual care and intervention group collected information in different ways. Persons with PD from the usual care group collected information on treatment options in an unstructured way, e.g., from their neurologist and PD nurse specialist, the internet, brochures, a DBS television documentary and experiences of other PwPD. PwPD in the intervention group collected information mainly from the SDM intervention, the internet and experiences of other PwPD. Information collection processes had a possible influence on decision quality and are, therefore, reported in detail there.

The dispersed nature of available information, different formats of folders and brochures and their initial preference for one treatment option were identified as factors limiting their information collection process in the care as usual group. PwPD furthermore reported that the unbalanced nature of information resulted in missing information to equally weigh risks and effects of available treatment options, and they wished to have received more detailed information on all options. Participants’ personal wish to search for information also impacted their information collection process as most participants stated they were hesitant to search for information on the internet because of its perceived overrepresentation of negative experiences. Although not explicitly mentioned by PwPD, we encountered several cases where participants made a treatment choice while having important questions regarding their final choice unanswered.

“I would want clear insight into how each option works, including a clear risk analysis that says that if we go for it this could be the outcome. Now it was a little hallelujah, that’s what she [neurologist] said, and the risks were not discussed.” (64 yrs, M)

Participants in the intervention group collected information mainly from the Option grid™ and online PDA, the internet and experiences from other PwPD. Participants stated that the Option grid™ and website increased their knowledge and understanding of treatments other than their initial preferred treatment. When PwPD searched for additional information, their information collection process was commonly structured based on the items represented in the Option grid™. Additionally, the Option grid™ supported patients in ensuring they had not missed any important information to make an informed choice. One participant commented that he would not have been able to acquire the information as presented in the SDM intervention by himself.

“Well, initially I knew most about duodenal levodopa, way more than about apomorphine. So, I gained more understanding of which I thought, ‘ahh so that works like that’ or ‘I did not know that’.” (60 yrs, M)

#### The intended versus actual use of the SDM intervention during the decision process

The persons’ interactions with components of the intervention in the intervention group were captured in the qualitative theme *the value of the SDM intervention during the decision process*. Although 46% of the PwPD reported in the questionnaire to have received or searched for information via the Option grid TM and website, interviews and logging data (Supplement 3 table S2) revealed that all PwPD exposed to the Option grid™ read its information. Most participants used the Option grid™ and the supplementary online information with a focus on their (initial) preferred treatment or to gain more profound information on the selected treatment option (Supplement 3 Table S3). Additionally, PwPD used the Option grid™ and accompanying online decision aid as a summary of the information they received during consultations. Participants commented that the timing of deployment of the Option grid™ by professionals at the end of the consultation contributed to this way of utilization. Some PwPD reported that the patient decision aid allowed them to prepare for the consultation by reading the provided information, which improved their understanding of the treatment options. Participants stated that they mainly utilized the value clarification tool to reiterate their preference and/or knowledge with the aim to affirm their preferred choice.

“The consultation was not guided by this [Option grid ^TM^]. It was presented to finish the consultation, like ‘here you have it as a summarized overview’.” (51 yrs, M)

“Yes, and the intervention provides you with more information, so you can start the consultation, or the dialogue better.” (60 yrs, M)

Multiple participants reported that using the Option grid™ and website was stimulated by the easy accessibility. The primary reason for not using one of the components of the intervention was because they had already made a final choice or collected information from other sources and didn’t need extra information. One participant stated he did not access the website as the link was provided on paper, while he would have preferred an online, clickable link.

“That is what I like, to be able to grab it [Option grid ^TM^] quickly to repeat for myself what everything was. […] And you can also show it to someone else.” (65 yrs, M)

“No, I mostly based my choice on the consultations with the neurologist and PD nurse specialist, who also showed me its [treatment devices] methods, how it looked and what it is. And also based on conversations I had alongside the consultations, at home and with other people in my surroundings. That was more guiding.” (49 yrs, M)

### Context

#### Cognitive functioning

Results of the MoCA cognitive tests showed that considerable percentages of participants had (mild) cognitive impairment in both the usual care and intervention group (50% vs 46%, *p* = 1.000) (Table 1). Participants did not report any difficulties in using the online patient decision aid. The interviews revealed that some PwPD had trouble understanding the representation of treatment effects and risks. This led one participant to believe that the represented numbers were based on studies with 100 patients while another participant interpreted no improvement in quality of life as a deterioration of quality of life.

#### The impact of having an initial treatment preference

Most PwPD in both groups (16/19 in usual care group, 11/13 in intervention group) started the decision process with a preference for one treatment option. The participants’ initial preferences were mainly based on hearing experiences with a device assisted treatment from another person with PD. This preference resulted in a biased decision-making process with the initial preference of the participant steering it. Beside PwPD having an initial preference as the starting point of the decision process, also the neurologist usually started the decision process with a preference for one treatment option.

“Well, it was more that I started with the idea I got from tips from the [hospital], who said ‘don’t you think it is time to start thinking about DBS?’” (49 yrs, M)

In both groups, PwPD reported that their own information search mainly focused on collecting information on their preferred treatment or on the preferred treatment of the neurologist and less on other available options, due to their initial preference. This was also reported from the OPTION scores on the information exchange during consultations. Of the 16 participants with consultation recordings in the usual care group, two persons were already referred for DBS screening before the information exchange and discussion about all treatment options took place and seven other participants had limited information exchange due to either their own clear treatment preference or due to treatment experience or preference of the neurologist or PD nurse specialist. Audio-recordings of the eight participants in the intervention group revealed that five persons received unbalanced information. Information was given on all three device assisted treatments, but the time spent on each treatment and the level of detail was much higher for only one of the treatments, for the same reasons as in the usual care group. Additionally, interviews showed that persons’ initial preferences were sometimes so definite that they were not interested in hearing or discussing information on other options. The observations of the consultations in the intervention group showed when the Option grid™ was used during the consultation, the options were more often directly compared and more equally discussed even when they had an initial preference.

“Yes, we feel that it never was a choice because we already heard from one experienced patient how he felt. We went on the internet again and although we hardly knew about the other two options, we quickly looked at it knowing that is not what I want. To work with a pump outside my body at this age. I don’t want that.” (51 yrs, M)

#### Professionals’ experience with device assisted treatments

The recordings of the consultations revealed that the PD nurse specialists’ experience with the device assisted treatments influenced the information exchange. Most often one or two treatments were discussed more elaborately, and it was explicitly mentioned that they could not elaborate on the other option due to lack of experience.

#### Organizational factors

Important organizational factors that facilitated the implementation of the SDM intervention were the national stimulation of implementing SDM in clinical practice, and the Dutch Society of Neurology, who increased awareness for all device assisted therapies among their members. An important barrier for the implementation was the simultaneous development of a printed folder on advanced therapies of the Dutch Parkinson’s disease Patient Association, based on our patient decision aid, which was used by two participating centers, instead of the patient decision aid (Table 4). The understaffing of PD nurse specialists was an important limitation for the inclusion and therefore a barrier for the implementation of the study.

#### Barriers and facilitators to SDM

We identified several facilitators and barriers to SDM, displayed in Fig. 1. Alongside the general factors that we found, factors that are specific to PD or this decision process were revealed. PD-specific facilitators were treatment devices of all device assisted treatments being shown to PwPD during the consultations, and the involvement of the PD nurse specialist in the decision process. PD-specific barriers included the opportunity to consult a person with PD who already underwent one of the device assisted treatments, as this completely steered the decision and emphasized only one option either negatively or positively based on that one person’s experience. The other PD-specific barrier perceived by PwPD themselves was the decreased decision-making capacities due to PD (Table 6).

**Table 6:** Overview of identified barriers and facilitators that influenced PwPD’s ability to participate in SDM

	Patient-professional interaction	Health care level	Contextual factors
Facilitators	Good relationship Discussing important treatment goals Being informed on all treatment options (despite preference) Neurologist adapts explaining to level of individual patient Neurologist empowers person with PD to make own choice without pressure Person with PD and professional share preferred treatment Being able to take preferred role	Being able to discuss decision with multiple professionals Easy-accessible communication with professional Multiple consultations for decision process Being aware of decision process outline Taking time for consultations and decision process	Person with PD feels some degree of urgency for decision process Support form partner/family
Barriers	Person with PD assumes that active role is not desired by professional Not being able to take preferred role Not discussing important treatment goals Initial preference limits information exchange Person with PD not really open for treatments other than preferred one		Presence of cognitive impairment Need for a fast change in treatment, rushes decision process
PD specific facilitators	Treatment devices shown during decision process	Involvement of PD nurse specialist in decision process	
PD specific barriers			Patient constructs preference in a biased way, based on the positive/negative experiences of other patients Decreased decision-making capacity in persons with PD

### Mixed methods analysis

**Fig. 2:**
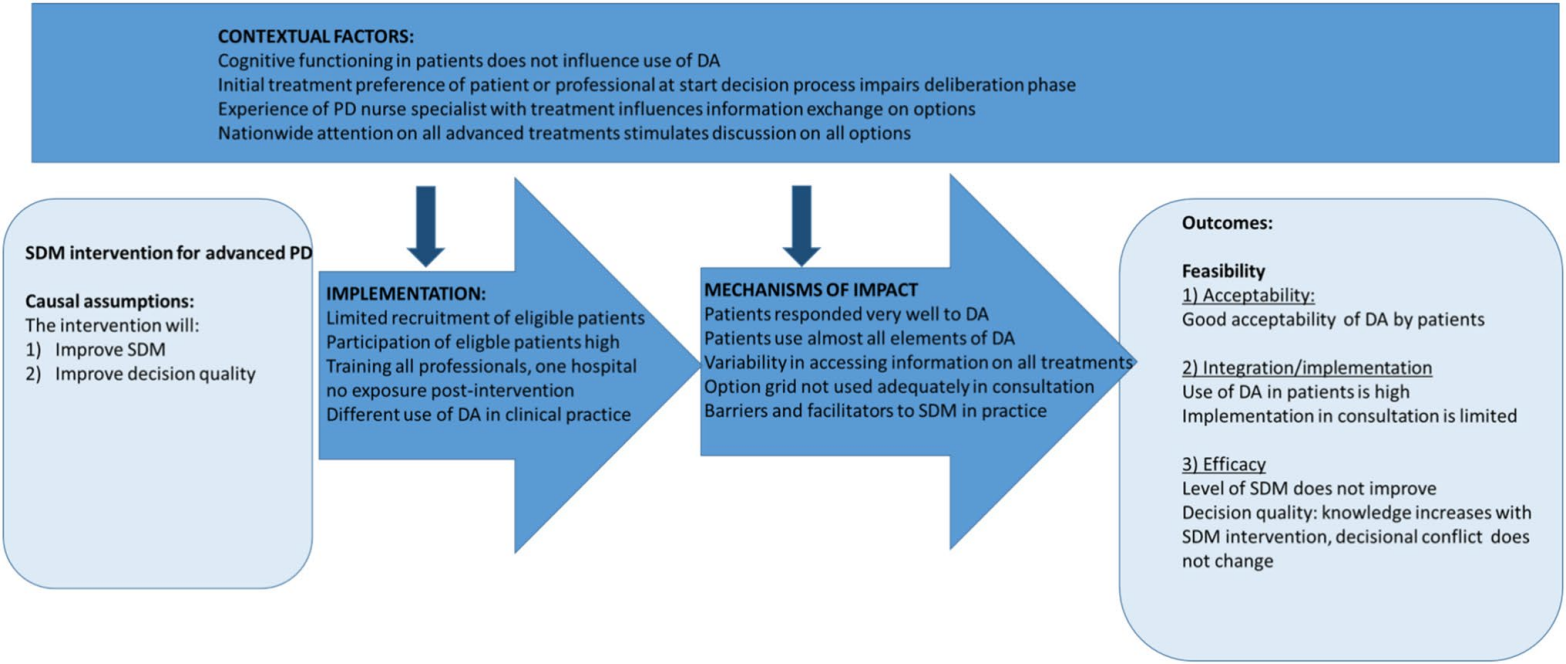
Triangulation of data on the implementation of the SDM intervention. *DA* decision aid

The objective of the SDM intervention was to increase the level of SDM and improve decision quality. The objective of this study was to evaluate feasibility of implementing the SDM intervention. Figure 2 represents the triangulation of all data to evaluate the feasibility of the SDM intervention. Assessment of the feasibility of the SDM intervention showed that recruitment of PWPD was difficult and even resulted in early termination of the study. Once PwPD were included, implementation of the SDM intervention overall was good with all but one person exposed to the SDM intervention. However, the Option grid™ was used differently in clinical practice than intended. The tool was handed out by the neurologists or PD nurse specialists, and the neurologists and PD nurse specialists subsequently used the tool; however, they used it merely to offer patients more background information, but less so as a tool to facilitate a joint deliberation together with the person with PD with regard to his or her preferences or rationale behind the choices. The acceptability of the PDA was good and knowledge of PwPD increased, while SDM did not improve combining all our data suggested that SDM did not improve due to a lack of using the Option grid™ during the consultations to have a deeper conversation on all treatment options and on the preferences/values of the PwPD. In other words, the Option grid ™ did not lead to a more in-depth deliberation on options and preferences. Another possible explanation is, that the initial preference of the PwPD or neurologist, or the experience with the treatments of the PD nurse specialist, hampers the shift towards SDM, not taking all three options into real consideration from the beginning. The lack of inclusion for the study itself was explained by the professionals by understaffing of PD nurse specialists, the lack of eligibility of PwPD, and an alternative (paper version) PDA.

## Discussion

Assessment of the feasibility of the SDM intervention showed limited implementation at the level of inclusion for the study, adequate levels of implementation at the patient level once included, and patients accepted both components of the SDM intervention; the Option grid™ and the online patient decision aid. Additionally, we found that the intervention increased patients’ knowledge on the various treatment options but this did not result in an improvement of the levels of SDM. Overall, these findings suggest that the SDM intervention is acceptable for PwPD and has the potential to support PwPD in their decision process on device assisted treatment selection. However, it also shows that increasing knowledge among patients is not sufficient to achieve SDM [7]. Furthermore, to reach feasibility for implementation, barriers to inclusion should be examined in more detail.

Improved levels of knowledge in the intervention group could be explained by the fact that these participants had important information readily available within the Option grid™ and the website. Additionally, the dispersed and unequal way of retrieving information in the usual care group could have hindered PwPD to become fully informed on all treatment options and their characteristics. While inadequate information provision remains an important barrier to SDM in other studies [26, 27], as well as the notion that information comprehension and recall can be limited in patients and structuring this information can improve recall [28] we argue that the Option grid™ and the website can reduce this barrier and support PwPD in participating in the SDM process if provided at an adequate moment in the decision process. A higher percentage of PwPD in the intervention group knew all device-assisted treatment options in comparison to the usual care group, but there was no statistically significant difference (77% vs 53%), although this could be partly explained due to small numbers. But even so, one would ideally strive for a 100% score for this specific knowledge item. Part of the lower score was explained by the fact that the initial preference steered professionals and patients to the preferred treatment, so consequently they did not gain extra information about the other treatments. That is why using the Option grid™ in the option talk is essential so that all options are discussed.

Despite improved levels of knowledge in the intervention group, we did not see any improvements on SDM scores. This observation can be explained by the high SDM-Q-9 and CollaboRATE scores in the usual care group, which, for the SDM-Q-9, has been reported by others as ceiling effects [29, 30]. The high baseline scores might be biased by PwPD feeling more important and involved in the decision process based on inclusion in this study or by social desirability bias, which could have masked improvements on the SDM-Q-9 and CollaboRATE measures [30, 31]. Additionally, during the inclusion period of this study, SDM received considerable attention in the Netherlands with a media campaign on the radio, tv, magazines and other public media channels [32]. This trend could have contributed to the relatively high SDM-scores found for the usual care group. Another explanation could be that professionals already altered behavior towards more SDM in the care as usual group, because they were aware of the objective of the study and were more conscious of their communication due to the fact the consultations were recorded [33]. Additionally, patients commonly do not know they are not informed on all options or that they have incomplete information [34].

Another explanation why SDM levels did not improve could be that the SDM intervention was not implemented as intended. Although the usage of the Option grid™ and the website at the patient level was adequate and comparable to other studies assessing the feasibility of SDM interventions [35, 36], implementation during consultation was inadequate. Interviews revealed that PwPD mainly used the Option grid™, website and value clarification tool as information source outside the consultation. Furthermore, PwPD suggested that the Option grid™ and value clarification tool were not used by the neurologist and/or PD nurse specialist, as a guide for discussing treatment options and person’s preferences, but as a handout of information or summary of options. Therefore, the intended aim of facilitating discussion during consultation and leading to more collaborative deliberation on preferences has not been achieved. This could have limited the utilization of the intervention’s potential to improve SDM, providing evidence that a conversation aid in the consultation is more geared to improve SDM if used as intended than a patient decision aid used by the patient autonomously [37].

Furthermore, SDM was possibly limited due to the initial treatment preference of the person with PD or the neurologist. The observations from the consultations, the logging data and the interviews revealed that having a strong initial preference for a treatment leads to a goal-directed search of information to confirm that preference. The person’s preference and the perceived preference of the neurologist can indeed be important predictors in treatment choice [2, 38]. Predicting these preferences has proven to be difficult [39]. The risk of misjudging each other’s preference is that you reach a shared decision, but the deliberation phase of the decision process is limited and does not entail checking whether the choice is congruent with personal values and preferences. In our study, the neurologists were more accurate in the intervention group in predicting the person’s preference at the end of the decision process. However, the interviews and observations did not show efforts made by the neurologist to challenge a patient on his or her preferences or to discuss elaborately on the construct behind the preference. If deeper discussion on preferences would take place, it could improve true collaborative deliberation [40] and through that route, SDM and decision quality improve, regardless of the final decision. Given the complexities involved in deliberating with a person with PD to weigh all facets of each therapy, it would ask for experienced professionals. This raises the question when a professional is experienced enough to discuss all treatments**.** The inclusion criteria stated that neurologist was eligible if he (1) considered at least five PwPD per year for aa device assisted treatment as part of the regular clinical practice. This is a very small number to represent ‘experience’ with device assisted treatments. It should be noted that the person with PD should be eligible for all treatments, and in clinical practice this excluded quite some persons, so professionals often see more PwPD that are eligible for one or two treatments. Furthermore, this was a minimum number, four of the five neurologists saw more than 20 eligible persons per year, and the final other one saw 10–20 eligible persons per year. The more important noted limitation in including PwPD was that often persons were referred for a device assisted treatment by another neurologist and were, therefore, already (partly) informed about treatment choices and were referred to receive a specific treatment. We observed in our previous studies [2, 27] on this decision-making process that PwPD were either referred immediately for a treatment (most often DBS) and did not receive information about the other treatment options. Also, once the person with PD arrived at the center of expertise with a specific request for a DBS procedure, the team at this center of expertise naturally assumed that the person had been referred to them after all options had been thoroughly discussed, and that DBS was already chosen deliberately out of all choices. It raises the valid question if these shared decision processes should only be performed in centers of expertise, where there is more time as well as expertise, with the condition that all the different treatment options are available. We acknowledge that deferring all shared decision processes entirely to centers of expertise that have all treatments available might lead to higher healthcare costs because of extra referrals (some of these initial costs might well be offset by later cost savings, because the patient receives the device assisted therapy that best matches the wishes and needs of all individual patients, which will likely lead to better compliance and possibly also greater efficacy).

Acceptability of the SDM intervention was good and use of the components of the decision aid were satisfactory, but the main barrier for a larger randomized trial would be the limited inclusion rates by professionals. The lack of inclusion for the study itself was explained by the professionals by understaffing of PD nurse specialists, the lack of eligibility of PwPD, and an alternative PDA that was used. The lack of eligibility could be reduced by training professionals when PwPD are suitable candidates for device-assisted treatments. The understaffing of PD nurse specialists is a realistic barrier in current healthcare settings. Although professionals did not mention this specifically as a barrier, it could be that time constraint was a reason they were not able to include patients. It is an important organizational barrier to consider if we would want to implement the SDM intervention in clinical practice. A method could be to simplify the SDM intervention, and only use the Option grid™ [14]. But our study confirms that training professionals is essential, otherwise the used decision support tool will not lead to SDM. Other studies have also confirmed that implementing SDM is much more complex [41, 42].

## Strengths and limitations

The major strength of this study is that we assessed the feasibility of the intervention both quantitatively and qualitatively, which allowed us to gain a profound understanding of the intervention’s acceptability and implementation from a patient’s perspective. The evaluation clarified why the intervention did not improve SDM as we expected, which gave direction for a modified intervention, putting more emphasis on the use of the patient decision aid in the dialogue between the patient and healthcare professional and creating a more detailed implementation training for new users.

Although we anticipated difficulties with the inclusion of PwPD in the study in our protocol [8] and included the criteria that neurologist/PD nurse specialist had to consider a minimum of five PwPD for device assisted treatment per year, we were unable to achieve the aim of including 20 PwPD in the intervention group. There is no consensus on a required sample size for pilot or feasibility studies, and numbers ranging from 10 to 100 participants per arm, with a median of 36 per arm for feasibility studies [43, 44], with a recommendation of 30 in total for qualitative feasibility studies. One center did not include any PwPD in the intervention group due to understaffing. Despite our efforts to improve inclusion rates by retraining the professionals, sending reminders and remove barriers for use (a computer mousepad with the steps of the study and in- and exclusion criteria, A4 tear-off pad of the Option grid), the limited numbers of inclusions in the intervention group have limited the strength of the statistical quantitative analysis. The small numbers of participants and included centers limits the external validity of our study. However, it is important to stress that this feasibility study explicitly intended to evaluate if the differences in setting (different levels of expertise, different availability of treatments) influenced feasibility. For instance, our observations of the consultations showed that information given with respect to the various treatment options varied greatly in length, depending on level of expertise for a specific treatment. The limited number of audio recordings of the observations could have limited the generalization of the OPTION scores as we do not have all data and the qualitative data gathered by the researcher from the observations (Table 5, last column) is limited. However, the qualitative analysis did give useful insights and all other data were not dependent on these recordings. The generalizability of the interviews was not compromised as data saturation for the qualitative interviews was reached. The limited inclusion does raise the question if there were other barriers that influence the implementation of the SDM intervention.

Furthermore, this study is in a certain level artificial as the intervention is introduced in a setting with PwPD who have a long-term relation with the neurologists and PD nurse specialists. This could have had two implications; first, the information retrieval did not start for patients with the start of this study and therefore the initial preference was already motivated well before our study started, because they considered other treatment options before, and already rejected some options. This could have led to the findings that the initial preference directed the decision process. We did however ask whether patients had been informed on options prior to the start of the study. Fourteen out of 19 (74%) in the usual care group and 8 out of 13 (62%) in the intervention group declared to not have been informed on options prior to the study and indicates that at least in majority of PwPD, the initial preference was not already motivated by prior information. Second, the included professionals knew that we introduced an intervention that should improve SDM at the start of the study. This could have changed their behavior in the decision processes in the usual care group already into more effort to perform SDM at baseline.

## Conclusion

The level of acceptability of the patient decision aid at the patient level was high, but the level of SDM did not improve. Important barriers to implementation included the lack of recruitment and professional’s lack of Option grid™ utilization in the consultation. Moreover, patients primarily used the intervention as information source, rather than as a basis for a joint discussion or deliberation with their healthcare professionals. These results indicate that attention should focus on stimulating patients and professionals to explicitly deliberate on preferences and coach the professionals in incorporating the Option grid™ and value clarification tool from the beginning in their consultations. In this way, SDM can be optimized during consultations where a decision for a device assisted treatment is made. Feasibility was not reached at the level of inclusion of PwPD that could benefit from this SDM intervention.

## Supplementary Information

Below is the link to the electronic supplementary material.

Supplementary file1 (DOC 297 KB)

Supplementary file2 (PDF 433 KB)

Supplementary file3 (DOCX 105 KB)
