# Supplementary material for: A decision aid is not the quick fix for improving shared decision-making in advanced Parkinson’s disease: results of a mixed methods feasibility study
Source: J Neurol. 2025 Mar 13;272(4):269. doi: 10.1007/s00415-025-12972-x (PMC11906552; doi:10.1007/s00415-025-12972-x)
Supplement: Supplementary file 1 — Supplementary file1 (DOC 297 KB) [file 415_2025_12972_MOESM1_ESM.doc]

**Supplement 1. Process evaluation of the SDM intervention**

To evaluate the SDM intervention, a complex intervention, a process evaluation is complementary to the outcomes evaluation. A process evaluation aims to understand the functioning of an intervention, by examining implementation, mechanisms of impact and contextual factors [1]. To create the framework for process evaluation we followed the guidelines of the MRC[1].To create the framework it is important to have a clear description of the intervention and to create a logic model which is diagrammatic representation of the relationships between an intervention’s resources, activities and intended outcomes [2]. The development of the SDM, as well as the full decision aid (PDF in Dutch) can be found through our data repository, DOI: <https://doi.org/10.34973/tfwt-0x10>. The framework for evaluation is based on the logic model and the anticipated effect of the SDM intervention. Figure 1 and 2 describe the evaluation process components such as the implementation process, mechanisms of impact, and the context.

The implementation process is evaluated by measuring fidelity, dose, adaptations and reach. Fidelity is the level of consistency of what is implemented. Dose represents how much of the intervention is implemented, adaptations are alterations made to the intervention, and reach is the extent to which targeted users are reached to use the intervention.

The mechanisms of impact are mechanisms through which the intervention activities produce intended or unintended effects. This can be evaluated by measuring the participants interactions with the intervention and measuring intermediate process that explain changes in outcomes. It is important to also evaluate unexpected pathways and their consequences.

The contextual factors are evaluated, which could be both factors specific for this decision process that influence the implementation of the intervention as well as factors that are affected by the intervention and which affect the intervention (figure 1).


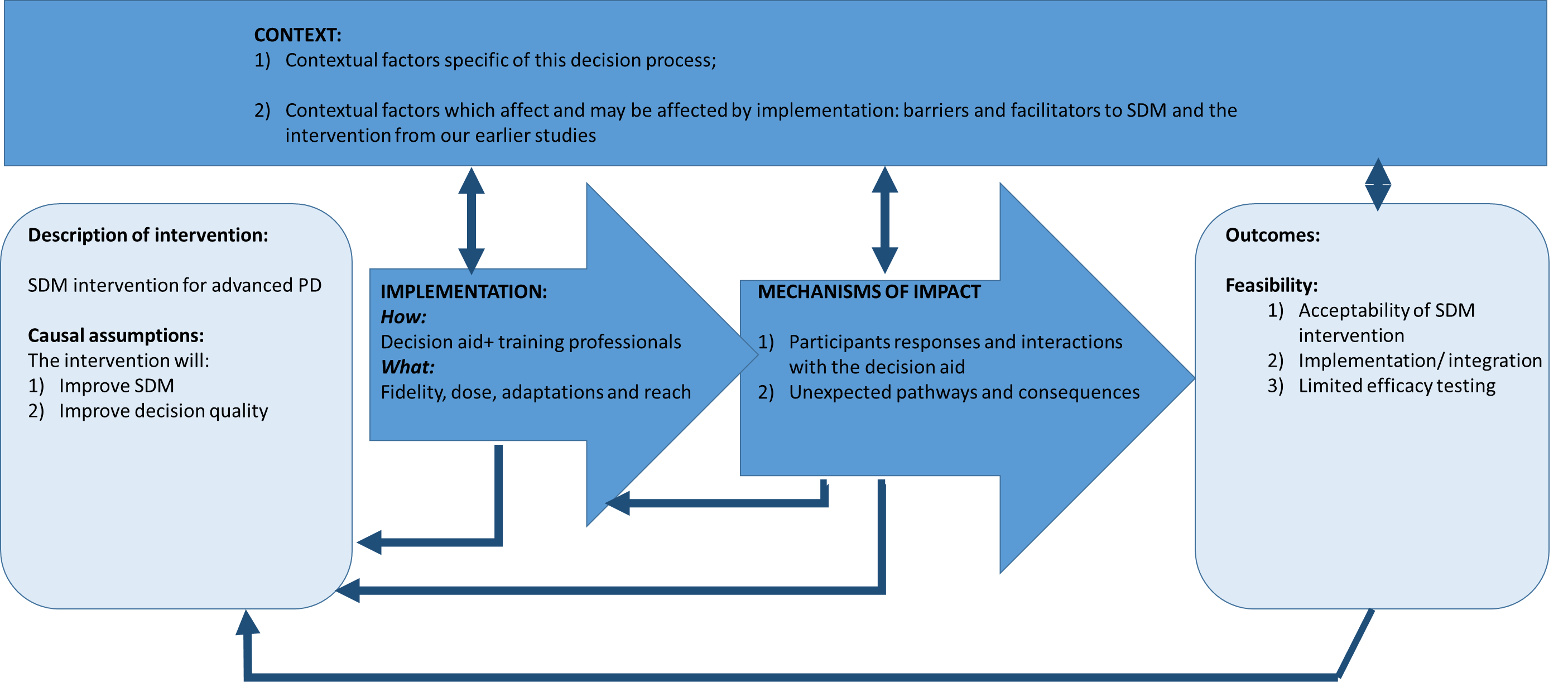


Figure 1: process evaluation framework concept for SDM intervention

In figure 2 the measurements are shown that will be used to evaluate the implementation process, the mechanisms of impact and the contextual factors. Using the triangulation protocol, these measurements will be combined to be able to analyze the acceptability and feasibility of the SDM intervention. Furthermore, having this evaluation process, the explanation of intended and unintended and expected and unexpected effects of the SDM intervention can be analyzed in more depth. In this article we report the data from the patient perspective.


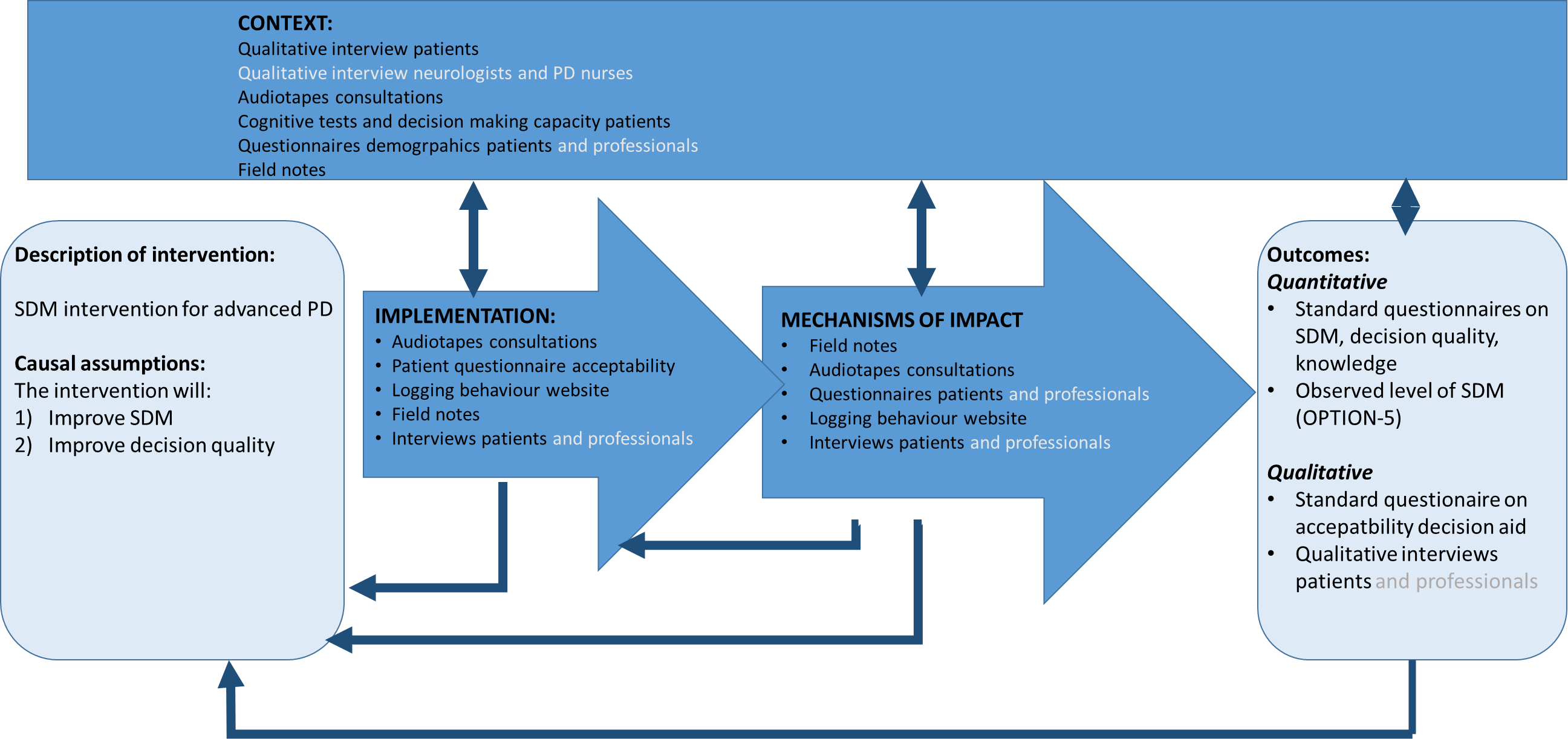


Figure 2. Process evaluation measurements for SDM intervention

OG = Option grid, DA = online decision aid, SDM= shared decision making

**Literature**

1. Moore, G.F., et al., *Process evaluation of complex interventions: Medical Research Council guidance.* BMJ, 2015. **350**.

2. Kellogg Foundation, W.K., *Logic model development guide* 2004, W.K Kellogg Foundation.: Battle Creek, MI.
