# Supplementary material for: A decision aid is not the quick fix for improving shared decision-making in advanced Parkinson’s disease: results of a mixed methods feasibility study
Source: J Neurol. 2025 Mar 13;272(4):269. doi: 10.1007/s00415-025-12972-x (PMC11906552; doi:10.1007/s00415-025-12972-x)
Supplement: Supplementary file 2 — Supplementary file2 (PDF 433 KB) [file 415_2025_12972_MOESM2_ESM.pdf]

Online Resource 2:

Supplementary material to *A decision aid is not the quick fix for improving shared decision-making in advanced Parkinson's disease: Results of a mixed methods feasibility study*

Journal of Neurology

Frouke A.P. Nijhuis*, Bas Schippers, Bastiaan R. Bloem, Bart Post, Marjan J. Meinders

* 1) Canisius Wilhelmina Hospital, Department of Neurology, Nijmegen, the Netherlands

2) Radboud university medical center, Research Institute for Medical Innovation, Department of Neurology, Nijmegen, The Netherlands.

Corresponding author at Radboudumc, Neurology 935, PO Box 9101, 6500 HB Nijmegen, The Netherlands. Tel: +31-622203266 Email: f.nijhuis@cwz.nl, ORCID ID: 0000-0003-1751-8650

Option Grid

The option grid was developed at a A4 format and was printed as a tear-off pad, that could be used in the consultation room. The option grid was developed in Dutch, but was translated to English for this publication. The additional online website, as well as the value clarification tool are also in Dutch. The numbers displayed in the option grid on the effects of each treatment in this version have been updated according to the last review we conducted [1]. The first version of the option grid had slightly different numbers but no significant differences.

1. Nijhuis, F.A.P., et al., *Translating Evidence to Advanced Parkinson's Disease Patients: A Systematic Review and Meta-Analysis.* Mov Disord, 2021. **36**(6): p. 1293-1307.

Overview of advanced parkinson treatments

parkinson decision aid

With this overview you can compare the three treatments with your current treatment. This is a brief overview of important questions about the treatments. For more information, please visit www.parkinsonkeuzehulp.nl

	Apomorphine pump	Deep brain stimulation	Duodenal Levodopa pump
**What is it?**	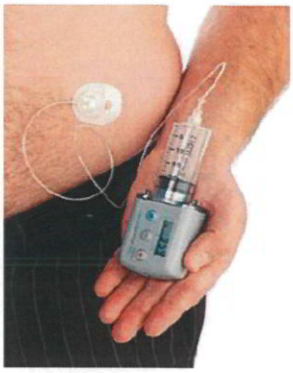<br>Apomorphine is administered continuously under the skin using a fine needle and pump system	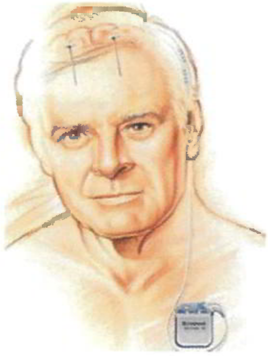<br>DBS is a procedure in which electrodes are placed that stimulate deep structures of the brain.	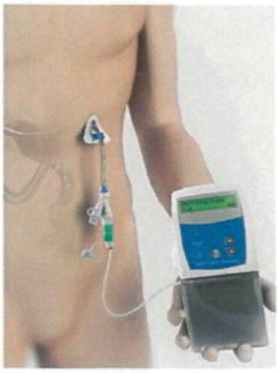<br>Levodopa is continuously administered via a tube in the small intestines and pump system

	Apomorphine pump	Deep brain stimulation	Duodenal levodopa pump
**What are the effects?**	**Quality of life**<br>34 of the 100 patients improve<br><br>**Daily activities**<br>gives an improvement not possible to give an estimate<br><br>**ON time**<br>3 hours more ON time per day	**Quality of life**<br>57 of the 100 patients improve<br><br>**Daily activities**<br>36 of the 100 patients improve<br><br>**ON time**<br>4 hours more ON time per day	**Quality of life**<br>62 of the 100 patients improve<br><br>**Daily activities**<br>44 of the 100 patients improve<br><br>**ON time**<br>4 hours more ON time per day

	Apomorphine pump	Deep brain stimulation	Duodenal Levodopa pump
**What are the risks**	Of 100 patients with apomorphine:<br><br>76 get skin problems (76%) of which 6 serious (6%)<br><br>11 get dizziness (11%)<br><br>17 get mood and/or behavioural problems (17%)	Of 100 patients with DBS<br><br>2 get cerebral bleeding (2%)<br><br>5 get speech problems<br><br>21 get mood and/or behavioural problems(21%)	Of 100 patients with duodenal levodopa<br><br>11 get a wound infection /abdominal infection (11%)<br><br>64 get pump problem (64%)<br><br>28 get mood and/or behavioural problems(28%)

	Apomorphine pump	Deep brain stimulation	Duodenal levodopa pump
**Do you still need pills?**	usually continue with Parkinson's tablets possibly a little less tablets	often fewer Parkinson's tablets than before sometimes the parkinson medication can be stopped	often the parkinson tablets can be stopped

	Apomorphine pump	Deep brain stimulation	Duodenal levodopa pump
**When are you not eligible?**	• daily care of pump not possible<br>• lsevere speech priver/ kidney problems<br>• severe dementia	• severe depression<br>• severe balance problems<br>• severe speech problems<br>• severe dementia	• daily care of pump not possible<br>• gastrointestinal disorders<br>• severe dementia

	Apomorphine pump	Deep brain stimulation	Duodenal levodopa pump
**What are the benefits?**	• relatively simple<br>• good effect on quality of life<br>• few contraindications<br>• treatment trial possible	• best studied<br>• good effect on quality of life<br>• often fewer tablets<br>• relatively less expensive	• reasonablywell studied<br>• good effect on quality of life<br>• no more tablets/ less tablets<br>• treatment trial possible

	Apomorphine pump	Deep brain stimulation	Duodenal levodopa pump
**What are the drawbacks?**	• less evidence<br>• external pump<br>• daily care needed	• operation required<br>• less people suitable<br>• no treatment trial possible	• operation required<br>• large pump to carry<br>• daily care needed

Questions/remarks after the consultation

Go to www.parkinsonkeuzehulp.nl for further information on all treatments
