# Supplementary material for: A decision aid is not the quick fix for improving shared decision-making in advanced Parkinson’s disease: results of a mixed methods feasibility study
Source: J Neurol. 2025 Mar 13;272(4):269. doi: 10.1007/s00415-025-12972-x (PMC11906552; doi:10.1007/s00415-025-12972-x)
Supplement: Supplementary file 3 — Supplementary file3 (DOCX 105 KB) [file 415_2025_12972_MOESM3_ESM.docx]

**Supplementary material supplement 3**

Table S1 .Acceptability ratings on different items of the decision aid

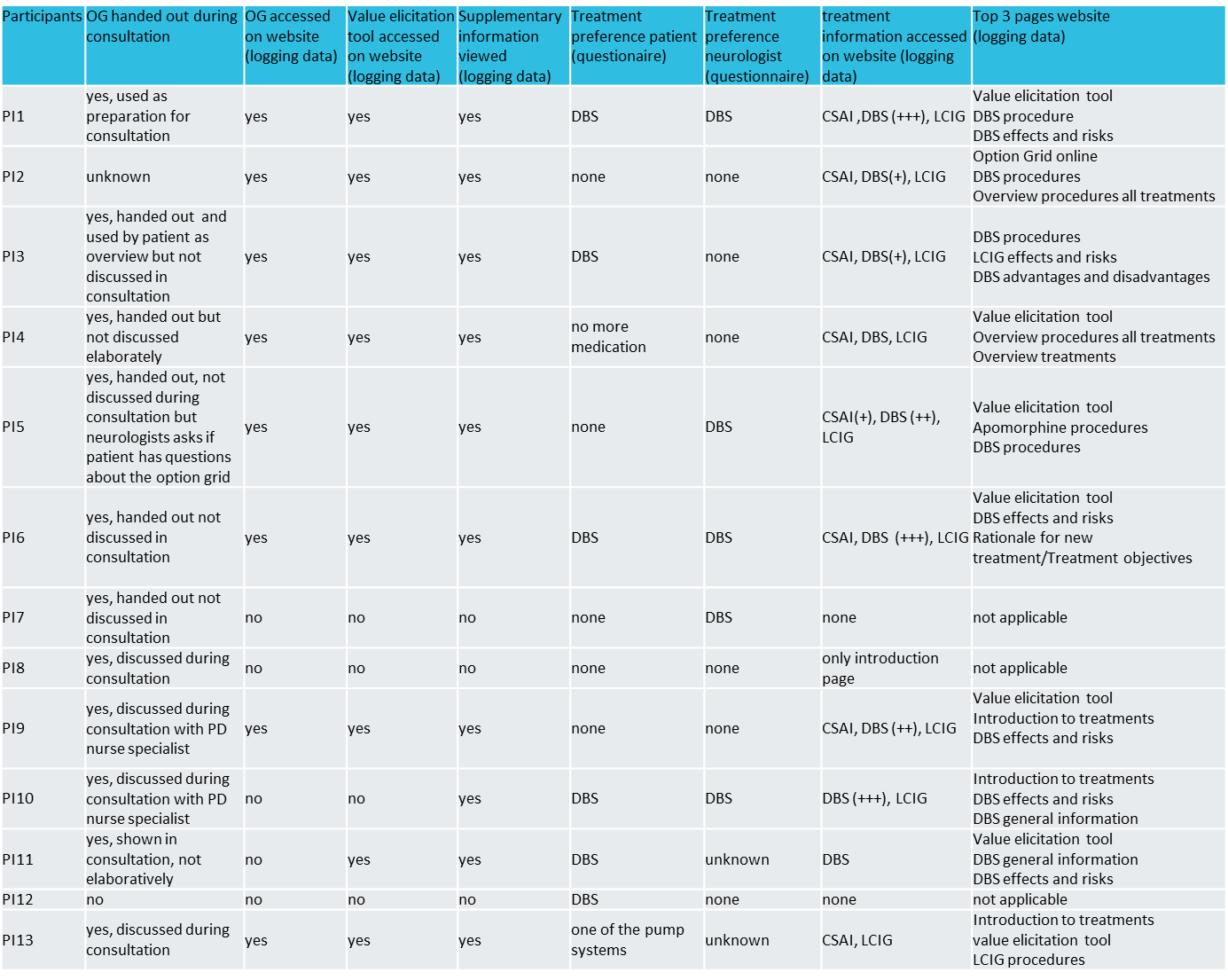
Table S2. Use of SDM intervention components

Table S3. Qualitative themes and sub-groups on implementation of the SDM intervention

| *Theme* | *Sub-group* | *Codes* |
| --- | --- | --- |
| ***The impact of having an initial preference*** | *Patient preference*  *Professional preference*  *Consequence of preference(s)* | Person with PD prefers or neglects treatment option after hearing experience of one patient.  Person with PD constructs preference through experiences of other patient(s) while being uninformed on other options.  Television documentary on DBS made participant prefer or neglect DBS as treatment option prior to the decision process.  Decision process is initiated with a preference for one treatment by the neurologist.  Initial preference limits profoundness of information need and search by participant. Person with PD not open for (information on) treatment options other than preferred one. Information in DA/consultation is filtered by participant due to having an initial preference.  Preference of person with PD and/or professional decreases completeness and profoundness of information exchange during consultation.  Not all options are discussed by professional due to preference of person with PD.  Neurologist attempts to improve information exchange, but preference of participant hinders this. Person with PD does not have access to all information due to preselection of treatment by professional. Person with PD feels professional is directing towards one option while person with PD is not ready for a decision yet.  Person with PD feels that professional emphasises information for preferred treatment. Patient's information search is directed by professional's preference.  Person with PD feels neurologist has different motivation for treatment choice (not patient centered). |
| ***The value of the SDM intervention during the decision process*** | *Patient’s utilisation of the DA*  *Facilitators and barriers to using DA* | Actual use of Option Grid and website by person with PD does not correspond to intended use by researchers.  Option Grid is used as summary for consultations.  Person with PD uses Option Grid and website to gain more profound information on preferred treatment .  Option Grid and website prepare person with PD for consultation.  Option Grid and website provides more realistic expectations for treatments for person with PD.  Person with PD is able to corroborate decision through Option Grid and website.  Participants can easily access information provided on the Option Grid.  Tear pad Option Grid provides participants with the opportunity to read printed version.  Option Grid and website creates opportunity to easily discuss information on options with partner and others.  Person with PD is unable to access online components in an easy way. |
| ***The patient’s search for information*** | *Pre-intervention group:*  *Post-intervention group:* | Profoundness of information provided on each treatment option differs. Treatment options are discussed in an unbalanced way during consultation. Not all aspects of preferred treatment by person with PD are discussed equally.  Person with PD did not receive complete information on the effects and risks.  Information from professionals on procedure after decision-making perceived as incomplete. Information from professionals on risks of treatment perceived as incomplete.  Information brochure is only provided for one treatment by professional. Different information formats provided for different treatment options.  Preference of person with PD and/or professional decreases completeness of information exchange. Person with PD wishes for balanced, unbiased overview of information on treatment options, but does not receive this.  Person with PD feels necessitated to search for additional information on the internet. Patient's own information search is directed by professional's preference.  Discussing all available treatment options despite patient’s/profesional’s preference.  DA increases knowledge and understanding of treatments other than initial preferred treatment. DA helped person with PD to collect information from one place.  Person with PD searches for information on treatment options on specific topic based on what he/she read in the DA. |

Table S4. Preferred versus perceived roles using the Control Preference scale

|  | **Preferred role** | | **Experienced role** | |
| --- | --- | --- | --- | --- |
|  | **Care as usual**  **[n (%)]** | **Intervention**  **[n (%)]** | **Care as usual**  **[n (%)]** | **Intervention**  **[n (%)]** |
| **Patient alone** | 4 (21) | 1 (8) | 6 (32) | 3 (23) |
| **Patient, considering neurologist’s opinion** | 6 (32) | 6 (46) | 7 (37) | 7 (54) |
| **Shared** | 9 (47) | 3 (23) | 3 (16) | 2 (15) |
| **Neurologist, considering patient’s opinion** | 0 | 2 (15) | 2 (10) | 1 (8) |
| **Neurologist alone** | 0 | 1 (8) | 1 (5) | 0 |

Table S5 . SDM-Q-9 scores and CollaboRATE subscores

|  | **Care as usual**  **[median (IQR)]** | **Intervention**  **[median (IQR)]** | **P-value** |
| --- | --- | --- | --- |
| **Overall SDM-Q-9 score** | **73 (60-84)** | **73 (60-78)** | **0.821** |
| My doctor made clear that a decision needs to be made. | 2 (0-3) | 2 (2-3) |  |
| My doctor wanted to know exactly how I want to be involved in making the decision. | 3 (2-5) | 4 (1-4) |  |
| My doctor told me that there are different options for treating my medical condition. | 4 (4-5) | 4 (4-5) |  |
| My doctor precisely explained the advantages and disadvantages of the treatment options. | 4 (3-5) | 4 (3-5) |  |
| My doctor helped me understand all the information. | 4 (3-4) | 4 (4-5) |  |
| My doctor asked me which treatment option I prefer. | 5 (4-5) | 4 (3-5) |  |
| My doctor and I thoroughly weighed the different treatment options. | 4 (3-5) | 4 (3-5) |  |
| My doctor and I selected a treatment option together. | 3 (3-4) | 3 (3-4) |  |
| My doctor and I reached an agreement on how to proceed. | 4 (3-5) | 4 (3-5) |  |
| **Overall CollaboRATE score** | **85 (70-89)** | **89 (46-89)** | **0.677** |
| How much effort was made to help you understand your health issues? | 8 (7-8) | 8 (4-8) |  |
| How much effort was made to listen to the things that matter most to you about your health issues? | 7 (7-8) | 8 (5-9) |  |
| How much effort was made to include what matters most to you in choosing what to do next? | 8 (6-9) | 7 (4-8) |  |

Table S6. Decision quality scores including subscales of the Decisional Conflict Scale
